# Impact of One-Catheter Strategy with TIG I Catheter on Coronary Catheterization Performance and Economic Costs

**DOI:** 10.5935/abc.20190232

**Published:** 2019-11

**Authors:** Joan Costa-Mateu, Diego Fernández-Rodríguez, Kristian Rivera, Juan Casanova, Patricia Irigaray, Marta Zielonka, Eduardo Pereyra-Acha, Albina Aldomá, Fernando Worner

**Affiliations:** 1 Cardiology Department, University Hospital Arnau de Vilanova, IRB, University of Lleida, Lleida - Spain

**Keywords:** Cineagiography/methods, Cardiac Catheterization/economic, Radiation, Ionizing;, Fluoroscopy, Cost Savings/economic

## Abstract

**Background:**

Coronary angiography with two catheters is the traditional strategy for diagnostic coronary procedures. TIG I catheter permits to cannulate both coronary arteries, avoiding exchanging catheters during coronary angiography by transradial access.

**Objective:**

The aim of this study is to evaluate the impact of one-catheter strategy, by avoiding catheter exchange, on coronary catheterization performance and economic costs.

**Methods:**

Transradial coronary diagnostic procedures conducted from January 2013 to June 2017 were collected. One-catheter strategy (TIG I catheter) and two-catheter strategy (left and right Judkins catheters) were compared. The volume of iodinated contrast administered was the primary endpoint. Secondary endpoints included radial spasm, procedural duration (fluoroscopy time) and exposure to ionizing radiation (dose-area product and air kerma). Direct economic costs were also evaluated. For statistical analyses, two-tailed p-values < 0.05 were considered statistically significant.

**Results:**

From a total of 1,953 procedures in 1,829 patients, 252 procedures were assigned to one-catheter strategy and 1,701 procedures to two-catheter strategy. There were no differences in baseline characteristics between the groups. One-catheter strategy required less iodinated contrast [primary endpoint; (60-105)-mL vs. 92 (64-120)-mL; p < 0.001] than the two-catheter strategy. Also, the one-catheter group presented less radial spasm (5.2% vs. 9.3%, p = 0.022) and shorter fluoroscopy time [3.9 (2.2-8.0)-min vs. 4.8 (2.9-8.3)-min, p = 0.001] and saved costs [149 (140-160)-*€*/procedure vs. 171 (160-183)-*€*/procedure; p < 0.001]. No differences in dose-area product and air kerma were detected between the groups.

**Conclusions:**

One-catheter strategy, with TIG I catheter, improves coronary catheterization performance and reduces economic costs compared to traditional two-catheter strategy in patients referred for coronary angiography.

## Introduction

Coronary angiography is the “gold standard” technique for the evaluation of coronary arteries.^1^ Due to its invasive nature, coronary angiography is associated with multiple complications. However, the rate of complications of coronary angiography procedures has decreased over time.^1^ Transradial access plays a key role by reducing vascular complications and mortality in patients undergoing invasive coronary procedures.^1-3^

Transradial access is currently the recommended strategy by clinical practice guidelines for coronary angiography.^1^ Nevertheless, there is no standard recommendation about the optimal coronary angiography strategy to perform these procedures. One-catheter strategy for radial coronary diagnostic procedures could help reduce radial spasm, complications related to contrast administration and exposure to ionizing radiation, since it avoids the exchange of angiography catheters during coronary procedures.^4-8^ However, despite the potential benefits, one-catheter strategy for coronary angiography by transradial access is not routinely used in many centres. This fact may be due, among other factors, to the need for operators to perform the learning curve or to the scarcity of data about its impact on catheterization performance and economic costs.

Therefore, the objective of our study is to compare two strategies: a one-catheter strategy with TIG catheters^6,9^ vs. a traditional two-catheter strategy with Judkins catheters, in order to determine if one-catheter strategy allows to reduce the amount of iodinated contrast, radial spasm, exposure to ionizing radiation and direct economic costs in diagnostic coronary angiography.

## Methods

### Population and Study Design

**Figure 1:**
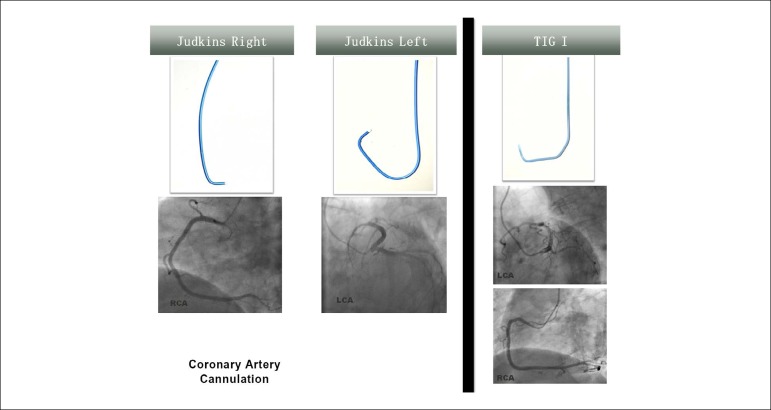
Design of TIG I and Judkins catheters and cannulation of the coronary arteries. RCA: right coronary artery; LCA: left coronary artery.

This study compared, in an observational and retrospective way, the impact of one-catheter strategy with TIG I catheter (Radiofocus Optitorque 5F; Terumo Europe N.V., Leuven, Belgium) and two-catheter strategy with Judkins catheters (Infiniti® 5F Left and Right Coronary Judkins; Cordis Corporation, Cashel, Ireland) on the amount of iodinated contrast, appearance of radial spasm, duration of the procedures and exposure to ionizing radiations in patients referred for diagnostic coronary angiography in our institution (Figure 1). We collected data on the procedures performed in our institution. For repeated procedures in the same patient, data of each coronary angiography were included separately. The study was conducted according to the principles of the Helsinki Declaration and in compliance with current ethical and legal regulations. All patients signed a written informed consent form before coronary catheterization.

Inclusion and exclusion criteria are described below:

* Inclusion criteria:
- Indication for invasive coronary angiography.
- Right radial access.
- Patients >18 years.
* Exclusion criteria:
- Abnormal Allen’s test.
- Presence of brachial arteriovenous fistula in right upper extremity.
- Need to use 4Fr catheters.
- Previous coronary artery bypass grafting surgery.
- Performance of ventriculography, aortography or ad hoc coronary angioplasty.
- Iodinated contrast allergy previously known that cannot receive pre-medication.
- Women with possibility of being pregnant.
- Inclusion in other clinical trials or registries.

* Endpoints and Definitions:

Primary endpoint was total volume (mL) of iodinated contrast used during diagnostic coronary procedures.

Secondary endpoints were related to the development of radial spasm, duration of the procedure, exposure to ionizing radiation and economic costs.

Radial spasm was defined as the presence of at least two of the following criteria: a) catheter manipulation resistance; b) pain in the arm during catheterization; c) pain after catheter manipulation; d) pain after sheath removal or e) resistance during sheath removal.^4^

Duration of the coronary procedure was indirectly evaluated by fluoroscopy time (min).

Exposure to ionizing radiation was evaluated by dose-area product (DAP) and air kerma.

Economic costs, measured in € per procedure, were defined as the direct attributable costs to each strategy for coronary angiography, including the type and number of catheters, the drugs used in the cath lab, the fungible material and the amount of iodinated contrast used in each procedure.^10,11^ Economic costs related to material used for coronary angiography are described in Supplementary Material.

### Procedural issues

Only patients referred for diagnostic coronary angiography by right radial access were recruited, because TIG I catheters are not designed to perform coronary angiography by left radial access or femoral access.^6,9^ Patients were assigned to two-catheter or one-catheter strategy at the discretion of the interventional cardiologist.

Palpable right radial artery, as well non-pathological Allen’s test, were mandatory to perform right radial access. In order to minimize arterial spasm, sublingual diazepam (10 mg) was given 30 minutes before the administration of local anesthesia subcutaneously. Using the Seldinger technique, a 5 or 6 Fr hydrophilic radial Glidesheath was implanted (RADIFOCUS® INTRODUCER II; Terumo Europe N.V., Leuven, Belgium). Then, an intra-arterial bolus with 2 mg of verapamil and 50 IU/Kg of unfractionated heparin was administered. The radial glide sheath was removed immediately after the diagnostic procedure, and hemostasis was obtained by 4-hour compression with conventional compressive dressings.^12^

Standard J-curve 0.035 guide wire (Radifocus M; Terumo Europe N.V., Leuven, Belgium) was used for the insertion and exchange of catheters. In order to obtain optimal quality images by coronary angiography, minimum of 5 views for the left coronary artery and minimum of 3 views for the right coronary artery were taken. The contrast volume used was 7 mL at 3 mL/sec for the left coronary artery and 4 mL at 2 mL/sec for the right coronary artery. However, the amount of contrast in each injection and the final number of views for correct assessment of the coronary tree was at the operator’s discretion.

Low-osmolar iodinated contrast media [Xenetix 350 (Iobitridol; Guerbert Group, Villepinte, France)] in combination with robotic contrast injector ACIST CVi® (ACIST Medical Systems, Eden Prairie, MN, USA) was used to make contrast administration uniform. The images were acquired as follows: low-quality fluoroscopy at 7.5 images/sec for coronary cannulation and cinefluoroscopy at 15 images/sec for coronary views.

Data related to baseline clinical characteristics, indication for coronary angiography and angiographic characteristics (number of coronary vessels with stenosis >50%), volume of iodinated contrast, radial spasm, access crossover, need for supplemental catheters, procedural duration, direct economic costs and information regarding exposure to ionizing radiations were collected. In the case of failure to engage the coronary artery ostium, crossover to alternative strategy was performed. All data generated were collected prospectively and entered into a specific computerized database.

### Statistical analysis

SPSS Statistics 24.0 software package (SPSS Inc., Chicago, IL, USA) was used for data analysis. All p values ​​were evaluated in two tails, with p values < 0.05 considered statistically significant. Categorical variables were expressed as count (percentage) and were compared using the chi-square test. Continuous variables were explored for normal distribution using the Kolmogorov-Smirnov test. Normally distributed variables were expressed as mean (1 standard deviation) and non-normally distributed variables were expressed as median (interquartile range) and were compared using unpaired Student’s t-test or U Mann-Whitney tests as appropriate.

## Results

**Figure 2:**
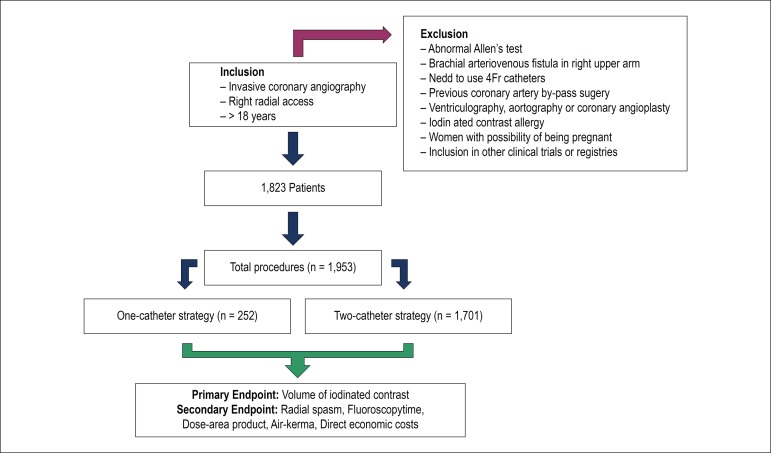
Study Flowchart.

A total of 1,953 diagnostic coronary procedures, in 1,829 patients, was collected between January 2013 and June 2017. Two-hundred fifty-two procedures (12.9%) were performed by one-catheter strategy and 1,701 procedures (87.1%) by two-catheter strategy. The study flowchart is shown in Figure 2.

### Baseline clinical characteristics

Baseline clinical characteristics are shown in Table 1. There were no differences between the two comparison groups

**Table 1:** Baseline clinical characteristics

	Total procedures (n = 1,953)	One-catheter strategy (n = 252)	Two-catheter strategy (n = 1,701)	p-value
Age, (years), mean (SD)	67.3 (11.8)	67.5 (12.1)	67.2 (11.6)	0.695
Female gender, n (%)	611 (31.3%)	82 (32.5%)	529 (31.1%)	0.645
BMI, (kg/m^2^), mean (SD)	28.0 (5.3)	28.1 (4.8)	28.0 (5.4)	0.657
Hypertension, n (%)	1443 (73.9%)	187 (74.2%)	1256 (73.8%)	0.858
Dyslipidemia, n (%)	1096 (56.1%)	142 (56.3%)	954 (56.1%)	0.861
Diabetes mellitus, n (%)	701 (35.9%)	98 (38.9%)	603 (35.4%)	0.288
**Smoking**				**0.539**
Non-smoker, n (%)	304 (15.6%)	39 (15.5%)	265 (15.6%)	
Previous smoker, n (%)	486 (24.9%)	56 (22.2%)	430 (25.3%)	
Current smoker, n (%)	1160 (59.5%)	157 (62.3%)	1003 (59.1%)	
Previous MI, n (%)	246 (12.6%)	27 (10.7%)	219 (12.9%)	0.426
Previous PCI, n (%)	318 (16.3%)	40 (15.9%)	278 (16.3%)	0.675
Family history of ischemic heart disease, n (%)	75 (3.8%)	11 (4.4%)	64 (3.8%)	0.669
Previous stroke, n (%)	65 (3.3%)	6 (2.4%)	59 (3.5%)	0.457
Chronic kidney disease, n (%)	223 (11.4%)	33 (13.1%)	190 (11.2%)	0.468
Creatinine (mg/dL), mean (SD)		1.15 (0.93)	1.07 (0.59)	0.647

SD: standard deviation; BMI: body mass index; MI: myocardial infarction; PCI: percutaneous coronary intervention.

### Procedural indications and angiographic characteristics

Data regarding angiographic indications and characteristics are shown in Table 2. No differences were detected between comparison groups in clinical presentation, diseased vessels and coronary artery disease extension.

**Table 2:** Procedural indications and angiographic characteristics

	Total procedures (n = 1,953)	One-catheter strategy (n = 252)	Two-catheter strategy (n = 1,701)	p-value
**Coronary angiography indication**				**0.684**
Chronic ischemic heart disease, n (%)	610 (31.2%)	74 (29.4%)	536 (31.5%)	
Acute coronary syndrome, n (%)	615 (31.5%)	77 (30.6%)	538 (31.6%)	
Valvular heart disease, n (%)	372 (19.0%)	49 (19.4%)	323 (19.0%)	
Myocardiopathy, n (%)	272 (17.1%)	43 (17.1%)	229 (13.5%)	
Other, n (%)	84 (4.3%)	9 (3.6%)	75 (4.4%)	
Left main coronary artery diseased, n (%)	155 (7.9%)	21 (8.3%)	134 (7.9%)	0.803
**Number of diseased vessels**				**0.359**
One vessel, n (%)	634 (32.5%)	83 (32.9%)	551 (32.4%)	
Two vessels, n (%)	294 (15.1%)	32 (12.7%)	262 (15.4%)	
Three vessels, n (%)	234 (12.0%)	25 (9.9%)	209 (12.3%)	

### Endpoints

Table 3 shows the comparative data regarding endpoints. The one-catheter strategy group received less amount of iodinated contrast than the two-catheter strategy group [77 (60-105) mL vs. 92 (64-120) mL; p < 0.001]. Also, the one-catheter strategy group presented less radial spasm (6.0% vs. 8.9%, p < 0.001) and shorter coronary procedures [Fluoroscopy time: 3.9 (2.2-8.0) min vs. 4.8 (2.9-8.3) min, p = 0.001] than the two-catheter strategy group.

**Table 3:** Endpoints

	Total procedures (n = 1,953)	One-catheter strategy (n = 252)	Two-catheter strategy (n = 1,701)	p-value
Volume of contrast, (mL), median (IQR)	90 (62-118)	77 (60-105)	92 (64-120)	< 0.001
Radial spasm, n (%)	176 (9.0%)	13 (5.2%)	163 (9.3%)	0.022
Access crossover, n (%)	92 (4.7%)	9 (3.6%)	83 (4.9%)	0.360
Supplemental catheters, n (%)	252 (12.9%)	40 (15.9%)	212 (12.5%)	0.132
Direct costs, (€/procedure), median (IQR)	169 (158-182)	149 (140-160)	171 (160-183)	< 0.001
DAP, (mGy.m2), median (IQR)	3685 (2408-5695)	3488 (2556-5369)	3711 (2393-5762)	0.831
Air kerma, (mGy), median (IQR)	630 (420-964)	582 (407-917)	641 (424-974)	0.165
Fluoroscopy time, (min), median (IQR)	4.7 (2.8-8.3)	3.9 (2.2-8.0)	4.8 (2.9-8.3)	0.001

IQR: interquartile range; SD: standard deviation; DAP: dose-area product.

No differences between one-catheter and two-catheter strategies were observed in access crossover (3.6% vs. 4.9%, p = 0.360) and need for supplemental catheters to complete coronary angiography (15.9% vs. 12.5%, p = 0.132). Also, there were no differences in exposure to ionizing radiations, evaluated as DAP [3488 (2556-5369) mGy.m2 vs. 3711 (2393-5762) mGy.m2; p = 0.831] and air kerma [582 (407-917) mGy vs. 641 (424-974) mGy; p=0.165].

Regarding economic analysis, the one-catheter strategy reduced direct costs attributable to coronary procedures [149 (140-160) €/procedure vs. 171 (160-183) €/procedure; p < 0.001] in comparison with the conventional strategy.

## Discussion

The main findings of our investigation were that one-catheter strategy, with TIG catheters, is associated with reduction in radial spasm, iodinated contrast consumption, duration of the coronary procedure and economic costs in coronary angiography.

Radial spasm is a relatively common complication during transradial coronary catheterization, and its incidence is variable, ranging from 5% to 30%.^12-16^ This complication reduces patient comfort and procedural success,^1-13^ and when it involves the need for crossover to transfemoral access it is related with an increase in vascular complications.^17^

Although the overall rate of radial spasm in our investigation (9.0%) was in the lower range of studies that have evaluated this item in coronary procedures, one-catheter strategy allowed reducing the incidence of radial spasm (one-catheter strategy: 5.2% vs. two-catheter strategy: 9.3%, p = 0.022). These results are in line with three of the most recent randomized clinical trials, demonstrating a reduction in radial spasm by the one-catheter strategy.^7,18,19^

Many factors, such as age, female gender, multiple radial punctures and radial diameter, are related with radial spasm.^4,14-16,20^ Furthermore, exchange of catheters during transradial access has been linked to radial spasm induction, probably related to repeated stimulation of the radial artery.^4^ As a result, radial spasm is not only associated with patient discomfort, procedural failure and morbidity and mortality, but also with high difficulty handling coronary catheters. This leads to more radiological tests to achieve cannulation of the coronary ostia and, therefore, to an increment in fluoroscopy time and total amount of iodinated contrast.

Iodinated radiological agents are related with several complications, highlighting contrast induced nephropathy (CIN). CIN, affecting 1% to 33% of patients referred for invasive coronary angiography, is one of the most common causes of acquired renal failure in cardiology patients.^20-24^ The development of CIN after an invasive coronary procedure is associated with long hospital stay, marked increase in morbidity and mortality, as well as an increase in health costs.^22,24^

Classical studies have shown that iodinated contrast volume used in invasive coronary procedures is closely related to the onset of CIN.^21,23,26^ To date, most studies on CIN prevention have not focused on specific techniques for reducing contrast administration. Only a recent observational study has shown reduction in CIN secondary to a specific technique for decreasing contrast administration by using rotational coronary angiography.^27^ Therefore, savings with iodinated contrast by one-catheter strategy, as shown in our investigation and corroborated by multiple studies,^7-10,18,27^ could reduce CIN.

Studying the economic impact of medical interventions is crucial to assess the implementation of new diagnostic/therapeutic techniques. A small observational study evaluated economic costs related to the use of TIG I catheter in a one-catheter strategy compared with Judkins catheters in a two-catheter strategy.^28^ Nevertheless, that study only evaluated costs related to the consumption of coronary catheters. To our best knowledge, our investigation is the first one evaluating all direct economic costs attributable to the one-catheter strategy for diagnostic coronary interventions. Our results show that it is related to a significant reduction in economic cost per procedure. This fact is mediated fundamentally by three factors: a) use of fewer coronary catheters; b) reduction in radial spasm, which reduces the use of more doses of spasmolytic medication and facilitates completion of diagnosis without using supplementary catheters and crossover to another arterial access with consequent expenditure on material; and c) reduced consumption of radiological contrast.

Although savings per unit are low (22 €/procedure), the long-term impact could be very important. Also, reduced use of iodinated contrast and the decrease in radial spasm could also reduce the indirect economic costs derived from CIN and crossover to transfemoral access.

### Limitations of the study

Firstly, this study is an observational analysis with inherent biases. However, this is one of the largest studies to evaluate the impact of using a one-catheter strategy with TIG I catheters in invasive coronary procedures. Secondly, data refer to the population of our geographic area and, therefore, cannot be fully extrapolated to other geographic areas. Thirdly, the performance of ventriculography and the diagnostic coronary procedure protocol may differ between different cath labs. However, we consider that our coronary angiography protocol can be considered conservative in the administration of contrast by excluding ventriculography and limiting the number of angiographic views and the volume of iodinated contrast per angiographic view and, for these reasons, we consider that comparison groups were well-balanced and the final volume of contrast administered was not overestimated. Fourthly, the type of material used (sheaths and catheters), as well as the size of radial sheaths can influence the development of radial spasm. The absence of detailed data regarding the size of the radial sheaths is a limitation of our study. However, the use of hydrophilic sheaths and widely-used catheter trademarks allow our data to be extrapolated to other cath labs. Fifthly, the economic analysis contemplates only the direct costs of the diagnostic coronary procedure and they refer to the prices in our institution. Nevertheless, our study is the first one evaluating total direct economic costs related to one-catheter strategy for coronary angiography. Also, as the savings are conditioned by the lower use of catheters and radial spasm and by the reduction in contrast administration, we consider that results could be easily transferable to other centres.

## Conclusions

The performance of diagnostic coronary angiography using the one-catheter strategy, with TIG catheters, was associated with better performance, in terms of radial spasm, administration of iodinated contrast and economic savings in diagnostic coronary procedures than conventional two-catheter strategy.

## Supplementary Material

**Direct attributable costs related to coronary angiography procedures (Prices of material used for coronary angiography)**

**Table:**

MATERIAL	UNIT PRICE
Catheter TIGER I RH-5TIG110M Radiofocus Optitorque® 5F; Terumo Europe N.V., Leuven, Belgium	17.45 €
Catheter 534518T INFINITI® 5F Judkins Left; Cordis Corporation, Cashel, Ireland	17.95 €
Catheter 534523T INFINITI® 5F Judkins Right; Cordis Corporation, Cashel, Ireland	17.95 €
Xenetix 350 100mL/Bottle; Guerbert Group, Villepinte, France	35.09 €
Sheath RT-R50G10PQ RADIFOCUS® INTRODUCER II 5 Fr or 6 Fr; Terumo Europe N.V., Leuven, Belgium	10.71 €
Exchange Guidewire M001491031 Starter "J" Curved Fixed Core 0.035 x 260 cm; Boston Scientific, Marlborough, MA, USA	8.40 €
Sterile Pack CombiSet® REF 266315; Hartmann, Heidenheim, Germany	40.31 €
Injector control AngioTouch® Kit; ACIST Medical Systems, Inc, Eden Prairie, MN, USA	38.00 €
Mepivacaine 2%; BBraun, Melsungen, Germany	0.87 €
Heparin 1%; Hospira Prod. Farm. y Hosp S.L, Alcobendas, Spain	1.89 €
Verapamil hydrochloride 2.5 mg/mL; Mylan, Canonsburg, PA, USA	0.59 €
